# Marburg virus outbreak in Tanzania: A threat to global health security

**DOI:** 10.1002/puh2.140

**Published:** 2023-11-20

**Authors:** Majani Edward, Godfred Yawson Scott, Withness John, Martha Ernest Rajabu, Elton Mahulu, Shuaibu Saidu Musa, Lucero‐Prisno III Don Eliseo

**Affiliations:** ^1^ Faculty of Medicine St. Francis University College of Health and Allied Sciences Morogoro Tanzania; ^2^ Department of Medical Diagnostics Kwame Nkrumah University of Science and Technology Kumasi Ghana; ^3^ Department of Nursing Science Ahmadu Bello University Zaria Nigeria; ^4^ Department of Global Health and Development London School of Hygiene and Tropical Medicine London UK; ^5^ Faculty of Public Health Mahidol University Bangkok Thailand; ^6^ Faculty of Management and Development Studies University of the Philippines Open University Laguna Philippines

**Keywords:** disease outbreak, global health security, infection control, Marburg virus disease, public health, viral hemorrhagic fever

## Abstract

The current Marburg virus (MARV) outbreak in Tanzania served as a stark reminder of the ongoing threat posed by emerging infectious diseases and the urgent need for global health security. The Tanzanian Ministry of Health (MoH) officially declared the outbreak on March 21, 2023. Eight cases in all, five of which included fatalities, have been reported in the country at present. The virus is a member of the Filoviridae family closely related to the widely known Ebola virus. Similar to other filoviruses, MARV causes acute and lethal hemorrhagic fever in both human and nonhuman primates with high case fatality rates ranging from 24% to 90%. The outbreak has highlighted the need for improved disease surveillance and response systems, as well as increased funding for research into emerging infectious diseases. The Tanzanian MoH has deployed a response team to investigate and monitor the transmission in the Kagera Region. The team works closely in collaboration with other organizations, such as the World Health Organization and the Africa Centre for Disease Control and Prevention, to ensure the effective control of the situation. Although there is no vaccine or treatment approved for Marburg virus disease (MVD), supportive management improves survival. Existing infection prevention and control protocols for Ebola and other viral hemorrhagic fevers such as isolation and use of appropriate personal protective equipment can be used to prevent transmission of MVD. The global community must work together to strengthen health systems, enhance research efforts, and build resilient and responsive health systems to prevent future outbreaks of this kind. In this article, we have analyzed the MVD outbreak in Tanzania, specifically in the Bukoba district of the Kagera Region, and provided recommendations for the management of the current outbreak and future outbreaks.

## INTRODUCTION

The deadly Marburg virus disease (MVD), caused by the highly pathogenic Marburg virus (MARV), has recently made headlines due to an outbreak in Tanzania, Africa. The outbreak was confirmed by the Tanzanian Ministry of Heath on March 21, 2023. A total of eight cases, including five deaths, were so far reported in the country [1]. Globally, a total of 669 cases of MVD have been reported as in March 2023 [2]. Since its discovery in 1967, the MARV has appeared in a number of African countries as shown in Table 1 [2]. Two major epidemics involving human‐to‐human transmission occurred in the Democratic Republic of the Congo and Angola, despite the fact that some of these cases were zoonotic [3].

**TABLE 1:** Distribution of Marburg by countries and years [2].

Country	Year	Total Cases	Symptoms
Kenya	1980	2	Fever, malaise
	1987	1	
South Africa	1975	3	Dense oropharyngeal secretions
Democratic Republic of the Congo	1998–2000	154	Fever, headache, nausea, vomiting, and loss of appetite
Angola	2004–2005	374	Fever, hemorrhage, cough, and diarrhea
Uganda	2007	4	Headache, musculoskeletal pain, nausea, vomiting, diarrhea, dyspnoea, fever
	2008	2	
	2012	15	
	2014	1	
	2017	3	
Ghana	2022	110	Fever, malaise, epistaxis, bleeding from the mouth, subconjunctival hemorrhage
Tanzania^a^	2023	8	Fever, vomiting, and bleeding from different body orifices

^a^ The most recent eight instances have been located in the most recent data for Tanzania, and five deaths, including a health‐care worker, have been reported (case fatality rate: 63%), whereas three are undergoing treatment at designated treatment centers [1].

The virus is a member of the Filoviridae family closely related to the widely known Ebola virus (EBOV) [4]. Similar to other filoviruses, MARV causes acute and lethal hemorrhagic fever in both human and nonhuman primates with high case fatality rates ranging from 24% to 90% [1, 4, 5]. Although MARV is typically 200 nm shorter than the EBOV, all members of the Filoviridae family are similar in size and range from 790 to 1400 nm long [6]. They have linear, negative‐sense single‐stranded RNA genomes and enclosed viruses with helical capsids [6]. The reservoir host of MARV is the African fruit bat, *Rousettus aegyptiacus* [7]. The incubation period of the virus ranges from 2 to 21 days [8].

The onset of symptoms is abrupt and is accompanied by fever, chills, headache, and myalgia [2, 8, 9]. A maculopapular rash may appear around the fifth day following the start of symptoms, with the chest, back, and stomach being the areas that are most noticeable [8, 9]. It is possible to experience nausea, vomiting, chest pain, a sore throat, abdominal pain, and diarrhea. Jaundice, pancreatic inflammation, extreme weight loss, disorientation, shock, liver failure, extensive bleeding, and multi‐organ malfunction are just a few of the symptoms that get progressively worse [9]. It might be challenging to make a clinical diagnosis of MVD as there are many similarities between the signs and symptoms of MVD and other infectious diseases (like malaria or typhoid fever) or viral hemorrhagic fevers that may be endemic in the area (like Lassa fever or Ebola) [9].

Between 5 and 7 days, many patients experience severe hemorrhagic symptoms, and fatal cases frequently involve numerous sites of hemorrhage [8]. Bleeding from the nose, gums, and vagina frequently accompany fresh blood in vomitus and feces [8]. At the sites of venipuncture (where intravenous access is established to administer fluids or draw blood samples), spontaneous bleeding can be particularly problematic [8]. Patients have experienced persistently high fevers during the acute stage of the illness. Confusion, impatience, and violence might result from central nervous system involvement. In the late stage of the illness (15 days), orchitis (inflammation of one or both testicles) has infrequently been recorded. Death in fatal instances typically happens 8–9 days after the onset of symptoms and is typically preceded by severe bleeding [8].

It is unknown how MARV first spreads from its animal host to people; however, for the two cases in tourists visiting Uganda in 2008, unprotected contact with infected bat feces or aerosols are the most likely routes of infection [7]. After this initial crossover of virus from host animal to people, transmission occurs through person‐to‐person contact. The virus spreads through contact (broken skin or mucous membrane) with blood or body fluids (urine, saliva, sweat, feces, vomit, breast milk, amniotic fluid, and semen) of an infected person, or contaminated objects such as clothes, bedding, needles, and medical equipment [4, 7]. There is no evidence that MARV can spread through sex or other contact with vaginal fluids from a woman who has had MVD [7].

## EPIDEMIOLOGY AND OUTBREAK OF MVD IN TANZANIA

**FIGURE 1:**
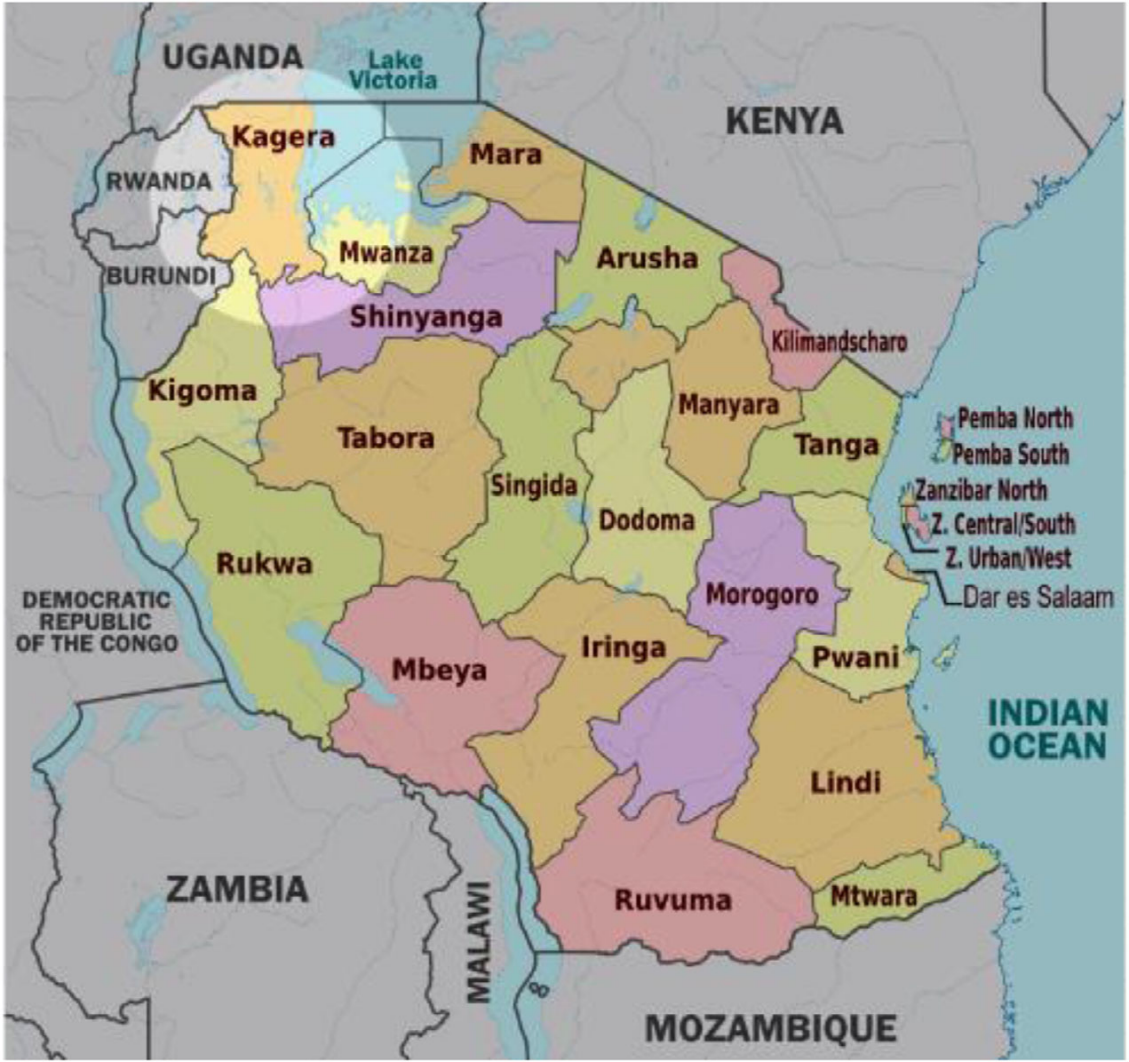
Map of Tanzania showing all the regions with focus on Kagera Region. The Kagera Region is highly relevant in this outbreak due to its geographical location and high trans‐border activities. The region shares borders with Uganda to the north and Rwanda to the west, making it region vulnerable to cross‐border transmission of infectious diseases like Marburg. *Source: Times* in Tanzania [11].

The first confirmed case of MVD in Tanzania was reported on March 21, 2023 by the Tanzanian Ministry of Health (MoH). The case was detected in Bukoba district, Kagera Region in north‐western part of the country as shown in Figure 1. This could result in cross‐border transmissions of the MVD as the Kagera Region is located in north‐western Tanzania, surrounded by Uganda to the north, Rwanda to the west, and Burundi to the southwest, further threatening the health‐care systems of these countries and Africa at large. Also, the high trans‐border activities within the region pose an additional risk of cross‐border transmission [1]. Although there is no vaccine or treatment approved for MVD, supportive management improves survival. Existing infection prevention and control protocols for Ebola and other viral hemorrhagic fevers such as isolation and use of appropriate personal protective equipment (PPE) can be used to prevent transmission of MVD in the country [1, 4, 10].

Following the outbreak of MVD in Tanzania, various efforts have been implemented to curb the spread of this fatal epidemic. The Tanzanian MoH has deployed a response team to investigate and monitor the transmission in the Kagera Region. The team works closely in collaboration with other organizations, such as the World Health Organization (WHO) and the Africa Centre for Disease Control and Prevention (Africa CDC), to ensure the effective control of the situation. The response team has implemented contact tracing activities to detect people with similar symptoms around the community. There is also improvement of safety measures for Health workers taking care of MVD patients to control infection around health‐care settings. The WHO's African region has also deployed an emergency team in Kagera to support the efforts of the MoH. The team is conducting further epidemiological investigations, including active case finding and providing appropriate care to those infected. This involves identifying individuals who have come into contact with MVD victims. WHO is working closely with local health authorities to ensure effective control of the outbreak by strengthening the surveillance system of the diseases [3].

In addition, the Africa CDC is supporting the country's immediate response efforts. This includes raising public awareness among the citizens about the disease to facilitate effective monitoring of cases. The Africa CDC is also assisting Tanzania and its neighboring countries in understanding the cross‐border context of the outbreak, which would aid in formulating regional surveillance strategies to combat the epidemic [1].

## RECOMMENDATIONS FOR FUTURE OUTBREAKS OF MVD

As far as the MARG outbreak in Tanzania is concerned, there is a need for improved health security in Africa and the world at large. It is important to improve surveillance systems to ensure the timely detection of infectious diseases outbreak. The capacities of medical laboratories in Tanzania and the rest of Africa need to be improved, which will enable early detection, accurate diagnosis, research, and effective response, ultimately reducing the impact of the outbreak on the population and contributing to global health security. WHO advises male MVD survivors to practice safer sex and hygiene for a full year after the onset of symptoms or until their semen tests negative for the MARG twice, based on additional analysis of ongoing research [4]. Health‐care workers need to be educated on the proper use of PPE and stringent infection control procedures to avoid becoming infected while caring for patients [4]. Safe burial practices are crucial to prevent transmission through contact with the deceased. This includes guidelines for handling and burying bodies safely, often with the involvement of trained personnel [12, 13]. Moreover, there is a need for continued investment in research and strengthening one health approach to develop new vaccines and treatments for infectious diseases [2, 14].

## CONCLUSION

The MARG outbreak in Tanzania is a wake‐up call on the importance of global health security in such a way that strong and resilient public health systems can be prepared to detect, prevent, and respond to the emergence of infectious diseases [15]. There are several key steps that can be taken to prevent similar outbreaks from occurring in the future. These include investing in improved surveillance, laboratory capacity, infection control measures, and strengthening research and one health approach for a safer and more secure global health system.

## AUTHOR CONTRIBUTIONS

*Investigation; validation; visualization; writing—original draft*: Withness John. *Conceptualization; formal analysis; investigation; software; supervision; validation; visualization; writing—original draft; writing—review and editing*: Majani Edward. *Conceptualization; investigation; writing—original draft*: Martha Ernest Rajabu. *Conceptualization; formal analysis; investigation; validation; writing—original draft; writing—review and editing*: Godfred Yawson Scott. *Investigation; writing—original draft*: Elton Mahulu. *Writing—review and editing*: Shuaibu Saidu Musa and Lucero‐Prisno III Don Eliseo.

## CONFLICT OF INTEREST STATEMENT

Shuaibu Saidu Musa is a Youth Editorial Board member of Public Health Challenges and a coauthor of this article. Don Eliseo Lucero‐Prisno III is the Chief Editor of Public Health Challenges and a coauthor of this article. They are therefore excluded from all editorial decision‐making related to the acceptance of this article for publication.

## FUNDING STATEMENT

The authors received no funding for this study.
